# High resolution identity testing of inactivated poliovirus vaccines

**DOI:** 10.1016/j.vaccine.2015.05.052

**Published:** 2015-07-09

**Authors:** Edward T. Mee, Philip D. Minor, Javier Martin

**Affiliations:** Division of Virology, National Institute for Biological Standards and Control, Medicines and Healthcare products Regulatory Agency, South Mimms EN6 3QG, Hertfordshire, UK

**Keywords:** Inactivated poliovirus vaccine, IPV, Vaccine contamination, Deep sequencing

## Abstract

**Background:**

Definitive identification of poliovirus strains in vaccines is essential for quality control, particularly where multiple wild-type and Sabin strains are produced in the same facility. Sequence-based identification provides the ultimate in identity testing and would offer several advantages over serological methods.

**Methods:**

We employed random RT-PCR and high throughput sequencing to recover full-length genome sequences from monovalent and trivalent poliovirus vaccine products at various stages of the manufacturing process.

**Results:**

All expected strains were detected in previously characterised products and the method permitted identification of strains comprising as little as 0.1% of sequence reads. Highly similar Mahoney and Sabin 1 strains were readily discriminated on the basis of specific variant positions. Analysis of a product known to contain incorrect strains demonstrated that the method correctly identified the contaminants.

**Conclusion:**

Random RT-PCR and shotgun sequencing provided high resolution identification of vaccine components. In addition to the recovery of full-length genome sequences, the method could also be easily adapted to the characterisation of minor variant frequencies and distinction of closely related products on the basis of distinguishing consensus and low frequency polymorphisms.

## Highlights

• Identity testing is a critical step in the quality control process.
• Serological testing is the current approved method, but has certain limitations.
• Existing molecular methods (qPCR) provide information about small genomic regions.
• Random amplification and shotgun sequencing provide full genome coverage.
• Distinction of highly similar viruses, and manufacturer-specific differences is possible.

## 1 Introduction

Rapid and accurate identification of poliovirus strains present in vaccines is important to ensure correct antigenic profile and limit the risk of an incorrect virus strain being included in a final product. The latter is a particular concern in cases where both attenuated (Sabin) poliovirus strains destined for oral and/or inactivated polio vaccine and wild-type strains destined only for inactivated vaccines or used as controls for quality control testing are handled on the same site.

Serological identification of vaccine components has been used effectively [1] and is the identification method approved in the European Pharmacopoeia [2], however this approach may be limited by the availability of appropriate reagents and inter-lab variation, may not distinguish between wild-type and attenuated strains of the same serotype and will not distinguish between virus strains that differ by a small number of nucleotides and/or amino acids. Resolution to the nucleotide level is useful not only in a prospective monitoring setting, but may also assist in tracing the source should a contaminant be detected. It also has the advantage that the nucleotide sequence of a given sample will be invariant regardless of the reagents and methods used for its determination. Several methods have been developed for identification of poliovirus strains at the molecular level, the most advanced of which was recently described by Nijst et al. [3]. This method, based on quantitative reverse transcriptase (RT)-PCR offers several advantages over serological testing including high specificity. It provides, however, information about only a small region of the genome (typically the 60–80 nucleotides constituting the primer and probe sequences), requires multiple reactions per sample to cover all possible strains and serotypes and has lower sensitivity for serotype 1 than for serotypes 2 and 3 [3].

Genome sequencing would provide the ultimate identity test. The availability of rapid, high-throughput benchtop sequencers makes full genome sequencing of multivalent vaccine samples feasible, with a tiny fraction of the time, effort and cost previously required. We evaluated a sequence-independent RT-PCR [4] and shotgun sequencing approach for the unambiguous identification of poliovirus strains present in a variety of inactivated poliovirus vaccines. All expected strains were detected in live and inactivated monovalent intermediates and inactivated trivalent final products. Retrospective testing demonstrated that the sequencing approach allowed for detection and characterisation of contaminating strains in an IPV product. This technique has the advantages of requiring only a single assay per sample, distinguishing between closely related strains and providing information on the full genome sequence of virus strains in a vaccine.

## 2 Methods

### 2.1 Vaccines

Samples were commercial vaccine components from different manufacturers, sampled at various stages of manufacturing: live monovalent samples, fomaldehyde-inactivated monovalent samples and trivalent formaldehyde-inactivated pools equivalent to the final vaccine product. Vaccine characteristics are described in Table 1.

### 2.2 Nucleic acid extraction

RNA was extracted using QIAMP vRNA Mini Spin columns or QIAMP MinElute Virus Spin Kit (Qiagen) without the addition of carrier RNA. One sample was extracted using the Kingfisher automated particle processor (Thermo Electron Corporation) and a MagNA Pure LC Total Nucleic Acid Isolation Kit (Roche), incorporating proteinase K digestion, following the manufacturer's instructions. Water only controls were extracted, amplified and sequenced in parallel with each set of samples.

### 2.3 Sequence-independent RT-PCR

Sequence-independent amplification was performed following the method of Victoria et al. [5]. cDNA was synthesised using the SuperScript III 1st strand cDNA synthesis kit (Life Technologies), with 8 μl RNA and 100 pmol primer K-N8 (5′-GAC CAT CTA GCG ACC TCC CAN NNN NNN N-3′), generating randomly primed first strand cDNA with an arbitrary 5′ tagging sequence. 10 μl of the first strand reaction was added to 50 μl second strand mix containing 100 pmol primer K-N8 (to randomly prime second strand synthesis and add the same arbitrary 5′ tagging sequence), 150 μM dNTPs (Roche) and 5 U Large (Klenow) Fragment DNA Polymerase in 1× REACT2 buffer (Life Technologies). The reaction was incubated at 37 °C for 1 h followed by 75 °C for 20 min, resulting in double-stranded cDNA with the tagging sequence ‘K’ at both ends. PCR was performed using 2.5 μl of cDNA in a 25 μl reaction containing 0.7 μM primer K (the arbitrary tag introduced during cDNA synthesis, 5′-GAC CAT CTA GCG ACC TCC CA-3′) and Kapa HiFi HotStart Ready Mix (Anachem). Amplification conditions were: 98 °C for 60 s; 30 cycles of 98 °C for 20 s, 60 °C for 30 s, 72 °C for 30 s; 72 °C for 5 min. Amplified products, comprising fragments of variable length and theoretically representing an unbiased sample of all nucleic acid in the sample, were visualised by ethidium bromide staining on agarose gels. A representative gel is shown in Supplemental Fig. S1. Products werepurified using AMPure XP magnetic beads (Beckman Coulter), quantified using Qubit High Sensitivity dsDNA assay (Life Technologies), analysed on an Agilent High Sensitivity DNA chip (Agilent) and diluted to 0.2 ng/μl in molecular grade Tris–EDTA, pH8.0.

### 2.4 Sequencing

Sequencing libraries were prepared using Nextera XT reagents (Illumina) and the manufacturer's protocol, and sequenced on a MiSeq using a 2 × 251 paired-end v2 Flow Cell (Illumina). Six to 10 vaccine samples were pooled for each run.

### 2.5 Primer, quality trimming and assembly

Raw sequence data were imported into Genious R7 (Biomatters) and paired end reads combined. Data were filtered and aligned using a custom workflow with the following parameters: shotgun primer K and Nextera adaptor/index sequences were trimmed from 5 and 3′ ends with a minimum 5 bp overlap; reads were trimmed to have an average error rate <1%, no more than one base with a quality of <Q20 and no ambiguities; reads shorter than 100 bases were removed. Reads were then mapped to references as defined in Table 2, with a minimum 50 base overlap, minimum overlap identity of 90%, maximum 10% mismatches per read, allowing up to 10% gaps, word length of 18 and index word length of 13. Reads with equally good matches to more than one reference were assigned randomly to one or the other. Results were displayed using Prism v5.02 (GraphPad Software).

### 2.6 D Antigen quantification

D Antigen content relevant of trivalent IPV products was determined using a standard control testing method [1].

### 2.7 Distinction of Mahoney and Sabin 1 strains

Reference consensus sequences for VP1 were obtained by mapping reads from live monovalent samples of known identity (90% consensus using highest quality base) against the VP1 region (nucleotide positions 2480 to 3385) of the Sabin 1 or Mahoney references described in Table 2. The Sabin 1 virus was derived from the parental Mahoney strain by repeated passage through monkeys and cell culture [6], and the reference sequences differ from each other by ∼57 nucleotides across the whole genome and 9 nucleotides in the VP1 region. The consensus sequence for Mahoney VP1 differed from reference strain (accession number V01149) at two positions in all vaccine viruses (nucleotide positions 2545 and 2626 of the whole genome sequence, corresponding to nucleotide positions 66 and 147 of the VP1 coding sequence). This modified sequence was used for subsequent analysis. For distinction between Mahoney and Sabin 1 strains, reads were assembled to the consensus VP1 coding sequence of the references in Table 2 using the assembly parameters described above. Consensus sequences were then extracted (90% consensus using highest quality base), aligned using Clustal W and examined to identify the serotype 1 component.

## 3 Results

### 3.1 Effect of virus inactivation on sequence read mapping

**Fig. 1:**
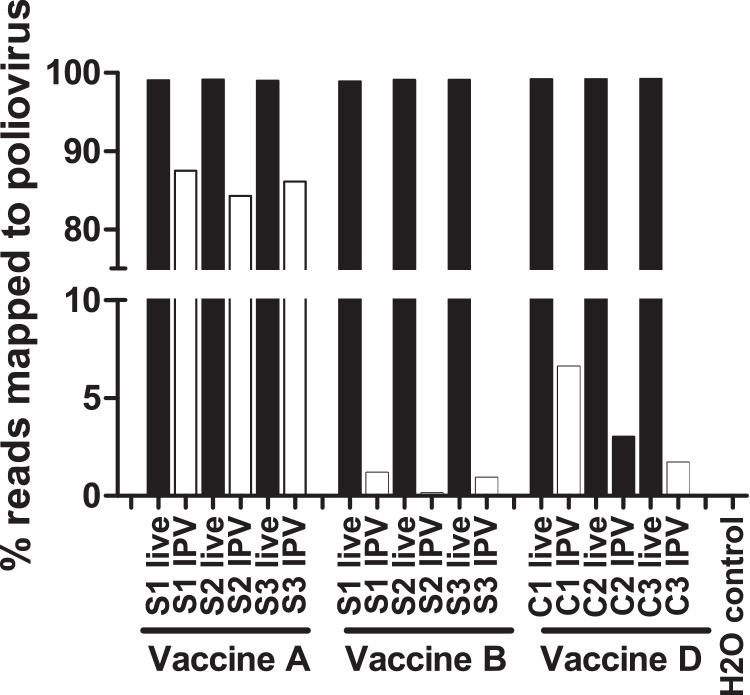
*Comparison of pre- and post-inactivation monovalent IPV samples.* RNA was extracted with QIAMP viral RNA Mini Spin Kit and random RT-PCR products sequenced and mapped to poliovirus reference strains. s Sabin, c conventional, *live* pre-inactivation monovalent virus, IPV inactivated monovalent virus.

We initially compared sequencing results from pre-inactivation and post-inactivation monovalent samples extracted using the QIAMP vRNA Mini Spin Kit. Pre-inactivation, greater than 99% of filtered reads mapped to the corresponding reference sequence. Post-inactivation, the percentage of filtered reads mapping to poliovirus was greatly reduced (range 0.14–87.5%), with clear differences between vaccine products (Fig. 1). The use of proteinase during RNA extraction was expected to improve recovery of poliovirus RNA; hence multiple extraction methods were compared.

### 3.2 Selection of RNA extraction method

**Fig. 2:**
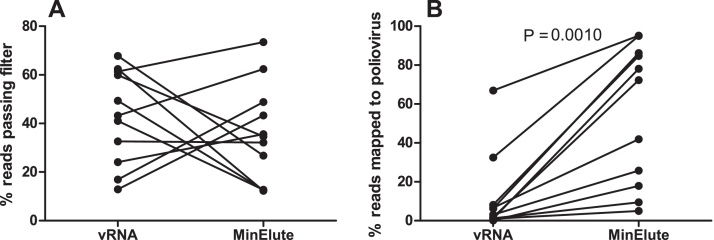
*Comparison of extraction methods for recovery of high-quality sequence data from IPV.* RNA was extracted using either QIAamp vRNA Mini Kit or QIAamp MinElute Virus Spin Kit and sequenced as described in methods. (A) Percentage of total reads passing trimming and quality filters. (B) Percentage of filtered reads mapping to poliovirus reference sequences. Methods were compared using a Wilcoxon signed rank test.

We compared sequencing results obtained with inactivated samples extracted using either the vRNA Mini Kit or the QIAMP MinElute Virus Spin Kit, the latter incorporating a proteinase K digestion step. No difference in overall sequence quality (assessed as percentage of reads passing adaptor/quality trimming) was observed between the kits (though it is noted that inter-run and inter-sample variability was high, Fig. 2A), however a much greater proportion of filtered sequencing reads mapped to the corresponding poliovirus reference in samples processed using the QIAMP MinElute Virus Spin Kit (*p* = 0.001, Wilcoxon signed rank test, Fig. 2B).

### 3.3 Sequence coverage of vaccines amplified by sequence-independent RT-PCR

**Fig. 3:**
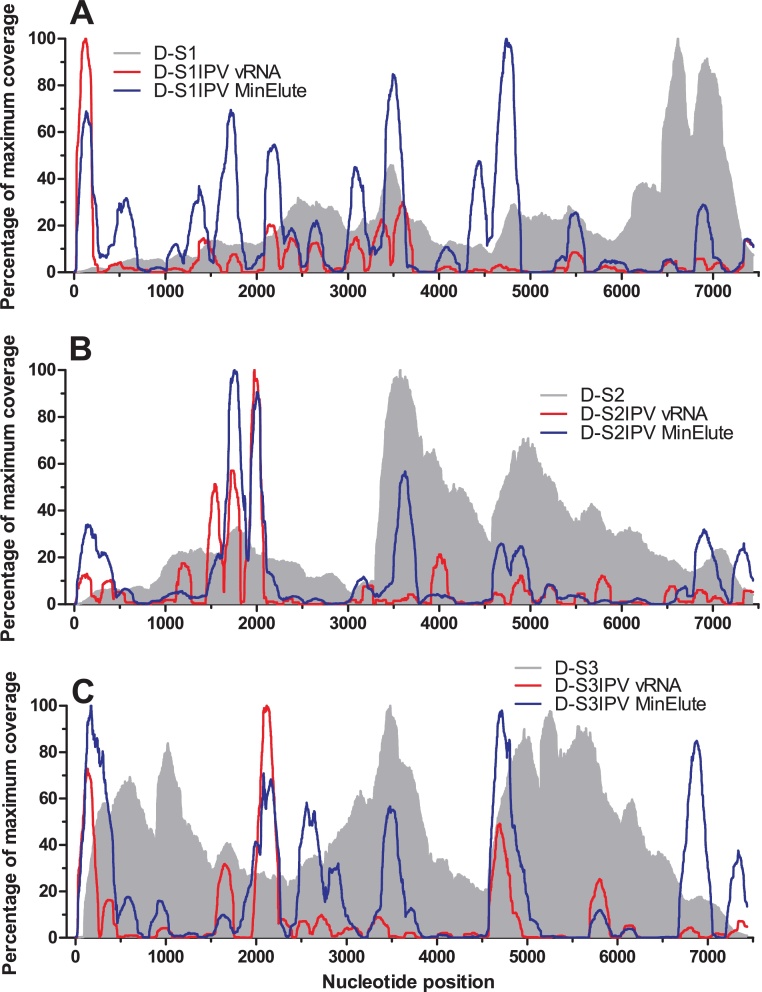
*Coverage of Sabin poliovirus genomes following random RT-PCR and sequencing of vaccine D*. Filtered reads from (A) Sabin 1, (B) Sabin 2 and (C) Sabin 3 monovalent samples were mapped against reference genome sequences and percentage of maximum coverage reported. *Grey* live virus, *red* inactivated sample extracted with QIAamp vRNA Mini Kit, *blue* inactivated sample extracted with QIAamp MinElute Virus Spin Kit. (For interpretation of the references to colour in this figure legend, the reader is referred to the web version of this article)

Depth of coverage for live poliovirus varied across the genome and between serotypes, consistent with other reports employing random RT-PCR amplification of total nucleic acid [4,7]. The most even coverage was obtained for live Sabin 3, while inactivated virus preparations were characterised by markedly uneven coverage for all three serotypes (Fig. 3). Coverage profiles did not appear to be affected by the extraction method.

### 3.4 Threshold for identity testing

**Fig. 4:**
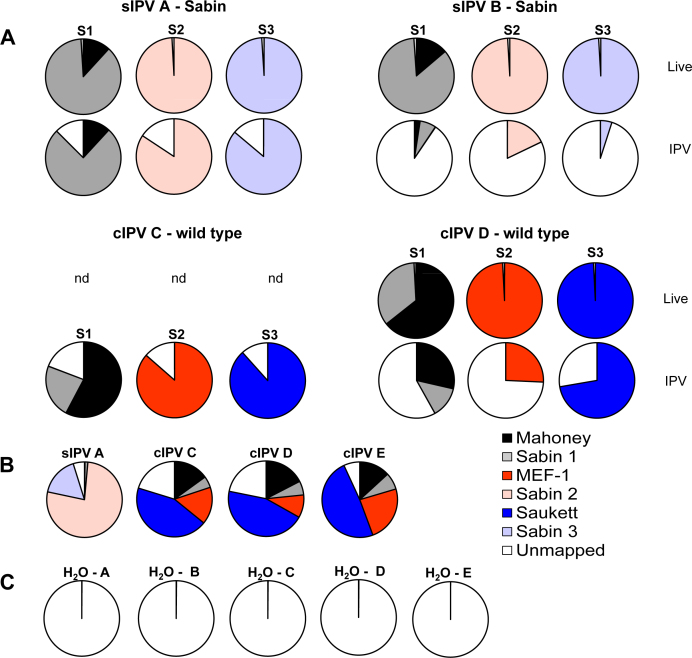
*Mapping and genome coverage of IPV samples*. Filtered reads from monovalent and trivalent samples of vaccines A–E were mapped against poliovirus reference sequences. (A) percentage of reads mapped to poliovirus references in monovalent live and inactivated samples. (B) percentage of reads mapped to poliovirus references in trivalent inactivated samples. (C) percentage of reads mapped to poliovirus references in parallel water controls. Letters indicate corresponding vaccine sample. nd Not done. Solid colours indicate wild-type virus, light colours indicate Sabin virus; white indicates unmapped reads. (For interpretation of the references to colour in this figure legend, the reader is referred to the web version of this article)

Following quality filtering, reads were mapped against all Sabin and wild-type poliovirus vaccine strains at high stringency (90% nucleotide identity with a minimum 50 nucleotide overlap). For all monovalent live viruses, the proportion of reads mapping against the expected genome sequence exceeded 90% (including aggregate counts for reads mapping to Sabin 1 and Mahoney) (Fig. 4). The proportion of reads mapped to the expected poliovirus sequence for the corresponding inactivated monovalent samples was lower and more variable (range 4.9–88% of filtered reads). For trivalent products, the proportion of reads mapping to each serotype varied between manufacturers (presumably reflecting the different concentration of individual viruses) but in all cases exceeded 1% of filtered reads. In no case did more than 0.1% of filtered reads map to unexpected poliovirus reference sequences.

### 3.5 Genome coverage of vaccine components

**Fig. 5:**
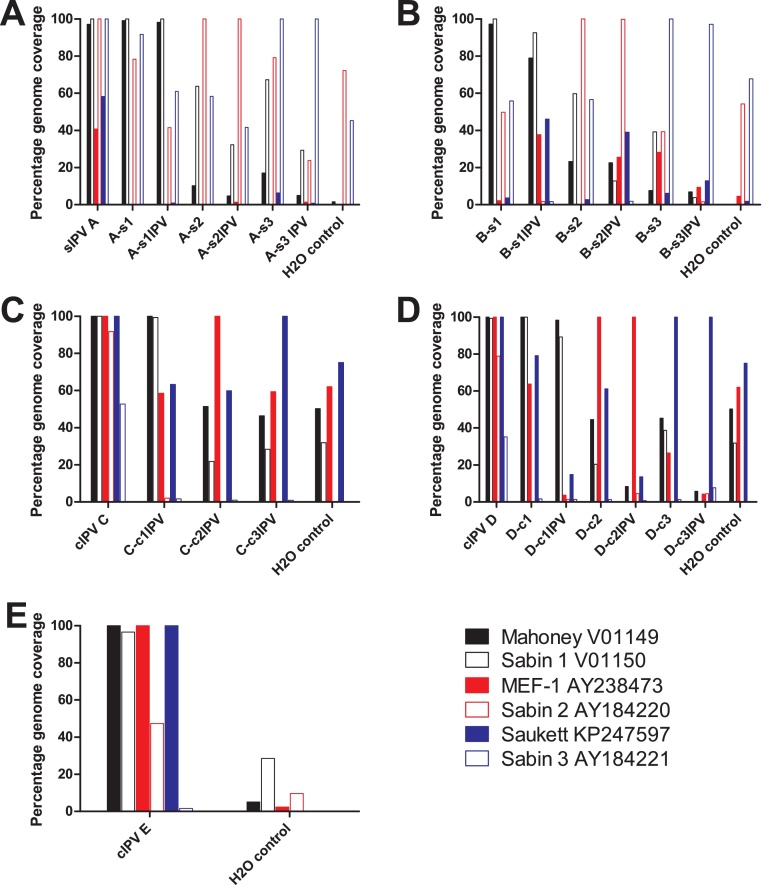
*Genome coverage of poliovirus references in IPV samples.* Filtered reads from monovalent and trivalent samples of vaccines A–E were mapped against poliovirus reference sequences. Graphs indicate percentage of indicated genome with ≥1× coverage. Solid colours indicate wild-type virus, light colours indicate Sabin virus. (For interpretation of the references to colour in this figure legend, the reader is referred to the web version of this article)

Typically, 100% (and in all cases greater than 90%) of the target genome was sequenced for expected vaccine components (Fig. 5) with depth of coverage ranging from 100 to 80,000 reads per nucleotide. In some samples, a small proportion of reads also mapped to unexpected poliovirus genomes. In some cases these reads covered large sections of the unexpected poliovirus genome, however low coverage (typically 0–5 reads per nucleotide) and large numbers of SNPs in the aligned regions (data not shown), indicated that these reads were mapped to regions of high similarity to the expected vaccine component rather than representing incorrect strains in the vaccine. The percentage of target genome sequenced alone did not therefore provide a reliable cut-off for assigning identity to a given vaccine component.

### 3.6 Distinction of Sabin 1 and Mahoney strains

The high degree of similarity (>99% nucleotide identity) between Sabin 1 and Mahoney strains of poliovirus resulted in large numbers of Mahoney reads mapping to the Sabin 1 reference sequence and vice versa. Increasing the stringency of mapping above 90% resulted in fewer reads overall mapping to both reference sequences but unambiguous mapping could not be achieved even at 100% identity (data not shown). To confirm the identity of serotype 1 viruses, consensus sequences were extracted from the VP1 region (containing nine distinguishing nucleotide differences) of mapped reads and aligned against the Mahoney and Sabin 1 reference sequences. In all cases, the consensus base at all nine positions matched the expected sequence (Table 3).

### 3.7 Correlation between number/proportion of mapped reads and D antigen content

**Fig. 6:**
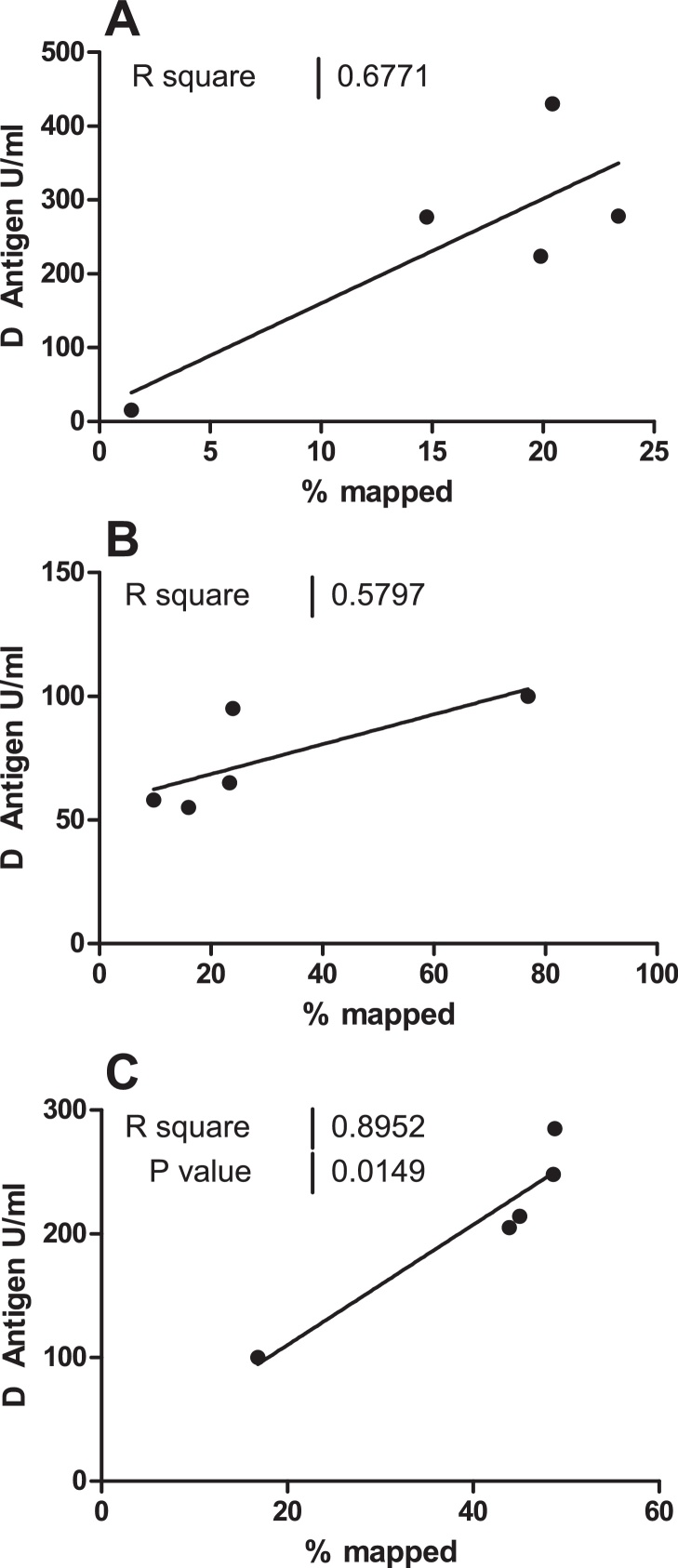
*Correlation between D Antigen content and percentage of reads mapping against poliovirus references.* D antigen content of vaccines was compared to percentage of reads mapped to each poliovirus reference using linear regression. (A) Serotype 1, (B) Serotype 2, (C) Serotype 3.

Potency data, based on D Antigen content, were available for all trivalent vaccines tested. Potency data were plotted against percentage of reads identified as each strain; a general trend for increasing percentage of reads mapped with increasing D Antigen concentration was apparent, however a significant correlation was observed only for serotype 3 (Fig. 6).

### 3.8 Detection of contaminating strains in a Sabin IPV vaccine

**Fig. 7:**
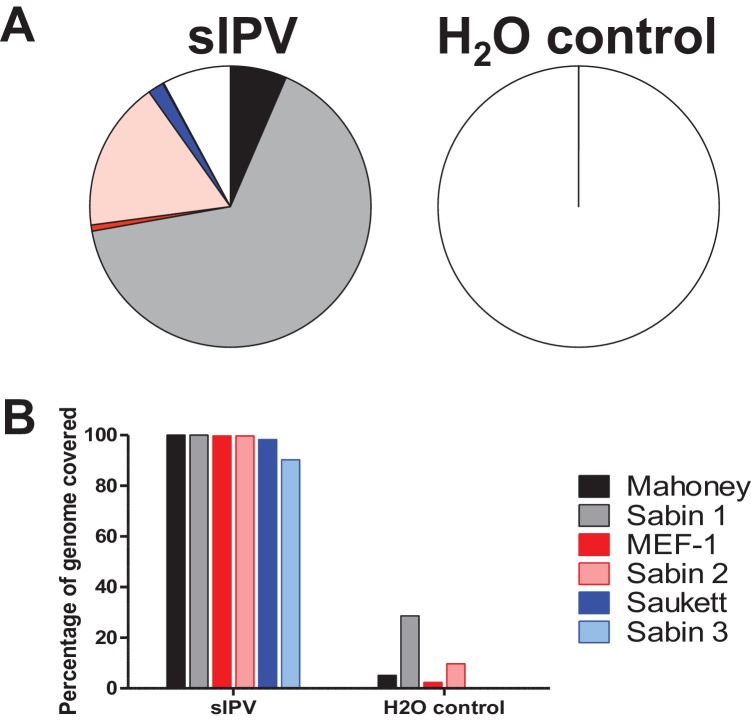
*Identity testing of a trivalent sIPV product.* Sample and water control were sequenced following random RT-PCR and reads mapped against poliovirus references. (A) percentage of filtered reads matching poliovirus references sequences. (B) percentage of reference sequence genome covered. Two wild-type strains (MEF-1 and Saukett) were detected in addition to the expected Sabin sequences. The VP1 consensus sequence for serotype 1 matched Sabin 1.

To evaluate the performance of the assay we analysed an inactivated Sabin poliovirus vaccine that was previously found to be contaminated with live Sabin 1 and inactivated wild-type strains of poliovirus. Previous investigations determined the presence of wild-type serotype 2 MEF-1, wild-type serotype 3 Saukett and live Sabin 1 as well as the expected inactivated Sabin strains (JM, unpublished data). The vaccine was at the Research and Development phase and was removed from any further pre-clinical development. At a threshold of 0.1% of filtered reads, the assay correctly identified Sabin 1-3, wild-type serotype 2 MEF-1 and wild-type serotype 3 Saukett (Fig. 7). Analysis of VP1 sequences confirmed the serotype 1 strain as Sabin 1 (Table 3), consistent with previous work (JM, unpublished data). Although this assay is not suitable for conclusively demonstrating the presence of live virus, coverage of the Sabin 1 was comparable to the profile of a live virus established previously (Fig. 3A and Supplemental Fig. S2).

### 3.9 Characterization of unmapped reads

A subset of unmapped reads for all samples were assembled and searched against the NCBI nt database; the majority of matches were to poliovirus or closely related coxsackievirus strains, with non-viral hits being predominantly human and bovine, likely reflecting the cells and serum using during propagation of the virus (data not shown).

## 4 Discussion

We have developed an identity test based on whole genome sequencing of inactivated poliovirus vaccines. The sequence-independent amplification approach employed here has the advantage of not requiring prior knowledge of the vaccine composition, does not require modification for different vaccine components and should not suffer bias or amplification failure due to sequence differences in primer binding regions. The method is more technically demanding than serological testing in its current format and will require further development and validation to surpass serological testing as the gold standard method for identity testing. Nevertheless, the considerable gain in the amount of information obtained, and likely reductions in assay time and complexity due to further advances in library preparation and sequencing technology suggest the method has potential to become a routine part of the quality control process for IPV.

Preliminary experiments using vRNA extraction columns and random RT-PCR revealed low and inconsistent coverage of inactivated samples. Formaldehyde treatment is known to reduce cDNA yields from inactivated poliovirus vaccines [8] and we reasoned that cross-linking of RNA to protein may prevent RNA from binding to the spin column. Differences between products may reflect varying degrees of RNA modification due to different particle:formaldehyde ratios during inactivation (JM and others, unpublished observations). The greater proportion of reads mapped when proteinase K digestion was used suggested that this method is preferable for recovery of RNA from inactivated poliovirus samples. Genome coverage of inactivated samples was uneven with no improvement with proteinase K digestion. Areas of high and low coverage may result from secondary structure and/or local RNA–protein interactions affecting the sensitivity of the RNA to formaldehyde treatment.

Expected poliovirus genome sequences were readily recovered from inactivated trivalent vaccines. Reads mapping to unexpected poliovirus genomes were typically located in regions of high similarity between wild-type and Sabin strains, and were readily apparent due to low and uneven genome coverage and the presence of large numbers of SNPs. A trivalent Sabin IPV previously found to be contaminated with live Sabin 1 and inactivated MEF-1 and Saukett was tested and both MEF-1 and Saukett were readily detectable. The expected Sabin 3 was present in only 0.1262% of reads reflecting the fact that the contaminating Saukett was present in greater abundance than the Sabin strain (JM, unpublished data).

Unambiguous mapping of reads to either Sabin 1 or Mahoney was challenging due to the high similarity between the two strains. By analysis of VP1 consensus sequences, the correct strain could be readily identified. This approach would not necessarily detect low level contamination of the serotype 1 component of the vaccine; however such contamination could be detected by variant calling within the mapped read set.

Reads in vaccines mapping to unexpected poliovirus genomes result from several causes. The simplest-similarity between Sabin and wild-type strains likely explains the vast majority of background reads. A higher mapping stringency would address this at the expense of a greater number of unmapped reads. Background reads in water controls are more of a concern but are explicable. The vast number of reads obtained by modern sequencing methods means that the detection of even trace levels of contamination occurring during sample extraction, amplification and library preparation is possible. Running and analysing parallel no-template controls from the extraction stage is therefore essential in every assay.

Correlation between D Antigen content and percentage of sequence reads mapped was poor, likely due to the nature of the assays: D Antigen is assayed for each serotype independently while proportions of sequencing reads mapped are interdependent. It is unlikely, therefore, that a sequence based approach would offer an alternative to D Antigen quantification.

Since the method recovers full-length genome sequence, the consensus sequence of each serotype can be compared against the reference and previous batches, as a measure of batch-to-batch consistency. Changing proportions of SNPs over time may also provide an indication of deviations within the production process. Both full-length consensus sequences and SNP profiles may also provide useful information about the source of a contaminant, should one be detected. In this study the consensus sequences of Sabin strains 1 and 2 and Saukett were invariant, but the Mahoney, MEF-1 and Sabin 3 strains contained ∼50, 5 and 4 positions, respectively, that varied between different products (data not shown) and from the reference sequence. Information about the potential source of environmental poliovirus, e.g. due to accidental release such as that occurring recently in Belgium [9] could also be obtained using this approach following the identification of contamination by routine screening methods.

A further development of the assay would permit screening for adventitious agents. Such assays require refinement, but the approach has already proven capable of detecting adventitious agents in a live attenuated vaccine [4]. Non-poliovirus sequences were detected in all samples tested in the current study. These included sequences of bovine and human origin, reflecting the host cells used for virus propagation and bovine viral diarrhoea virus and bacterial and eukaryotic hits likely arising from reagent contamination [10–12]. The lower efficiency of amplification from formaldehyde treated samples [8] may result in apparent over-representation of contaminating reads in such samples, however similar classes of non-poliovirus sequence reads were present in both live and inactivated samples.

In summary, we have demonstrated the utility of a method for identification of all poliovirus strains in trivalent inactivated vaccines (and upstream monovalent bulks) within a single assay. Mahoney and Sabin 1 strains could be distinguished and contaminants readily identified. The ability to reconstruct near full-length genome sequences even from inactivated products also provides important sequence information that may assist not only with detection of contaminants, but also with identifying or excluding likely sources. The assay can likely be adapted to identity testing of any nucleic-acid containing vaccine product.

## Author contributions

ETM, PDM and JM conceived and designed the study. ETM performed data collection and analysis. ETM, PDM and JM wrote, revised and approved the manuscript.

## Conflict of interest statement

The authors declare no conflict of interest.

## Figures and Tables

**Table 1:** Vaccine material.

Vaccine	Trivalent product	Monovalent product	Live/inactivated	Extraction
A	sIPV A		Inactivated	MinElute
		A-s1	Live	vRNA
		A-s2	Live	vRNA
		A-s3	Live	vRNA
		A-s1IPV	Inactivated	vRNA
		A-s2IPV	Inactivated	vRNA
		A-s3IPV	Inactivated	vRNA
B		B-s1	Live	vRNA
		B-s1IPV	Inactivated	MinElute
		B-s2	Live	vRNA
		B-s2IPV	Inactivated	MinElute
		B-s3	Live	vRNA
		B-s3IPV	Inactivated	MinElute
C	cIPV C		Inactivated	MinElute
		C-c1IPV	Inactivated	MinElute
		C-c2IPV	Inactivated	MinElute
		C-c3IPV	Inactivated	MinElute
D	cIPV D		Inactivated	MinElute
		D-c1	Live	vRNA
		D-c1IPV	Inactivated	MinElute
		D-c2	Live	vRNA
		D-c2IPV	Inactivated	MinElute
		D-c3	Live	vRNA
		D-c3IPV	Inactivated	MinElute
E	cIPV E		Inactivated	MagNA Pure
F	sIPVsample		Inactivated	MinElute

s Sabin; c conventional; IPV inactivated polio vaccine; vRNA QIAMP viral RNA Mini Spin Kit; MinElute Qiagen MinElute Virus Spin Kit; MagNAPure MagNA Pure LC total RNA isolation kit.

**Table 2:** Reference sequences used for assembly.

Serotype	Strain	Reference length	Accession number	Notes
1	Mahoney	7440	V01149	
	Sabin 1	7441	V01150	
2	MEF-1	7440	AY238473	
	Sabin 2	7439	AY184220	
3	Saukett	7429	KP247597	Full-length sequence constructed from live serotype 3 vaccine samples
	Sabin 3	7432	AY184221	

**Table 3:** Distinction of Sabin 1 and Mahoney strains by analysis of VP1 consensus sequences.

Sample	Sabin-specific positions^a^	Mahoney-specific positions^a^
sIPV A	9	–
A-s1	9	–
A-s1IPV	9	–
B-s1	9	–
B-s1IPV	9	–
cIPV C	–	9
C-c1IPV	–	9
cIPV D	–	9
D-c1	–	9
D-c1IPV	–	9
cIPV E	–	9
sIPV vaccine	9	–

a Based on 9 unique positions in the 906 nucleotide sequence of VP1.

## Appendix A Supplementary data

Fig. S1 Representative ethidium bromide-stained agarose gel showing randomly amplified DNA from poliovirus vaccines. (1–3) live monovalent Sabin vaccines, (4–6) inactivated monovalent Sabin vaccines, (7) water extraction control, (8) water control for cDNA synthesis, (9) water control for PCR, M: PhiX/HaeIII digest DNA size ladder. Gel has been cropped to remove unrelated samples.

Fig. S2 Genome coverage of Sabin 1 in contaminated sIPV product.
