# Supplementary figures and images for: High resolution identity testing of inactivated poliovirus vaccines
Source: Vaccine. 2015 Jul 9;33(30):3533–41. doi: 10.1016/j.vaccine.2015.05.052 (PMC4504004; doi:10.1016/j.vaccine.2015.05.052)

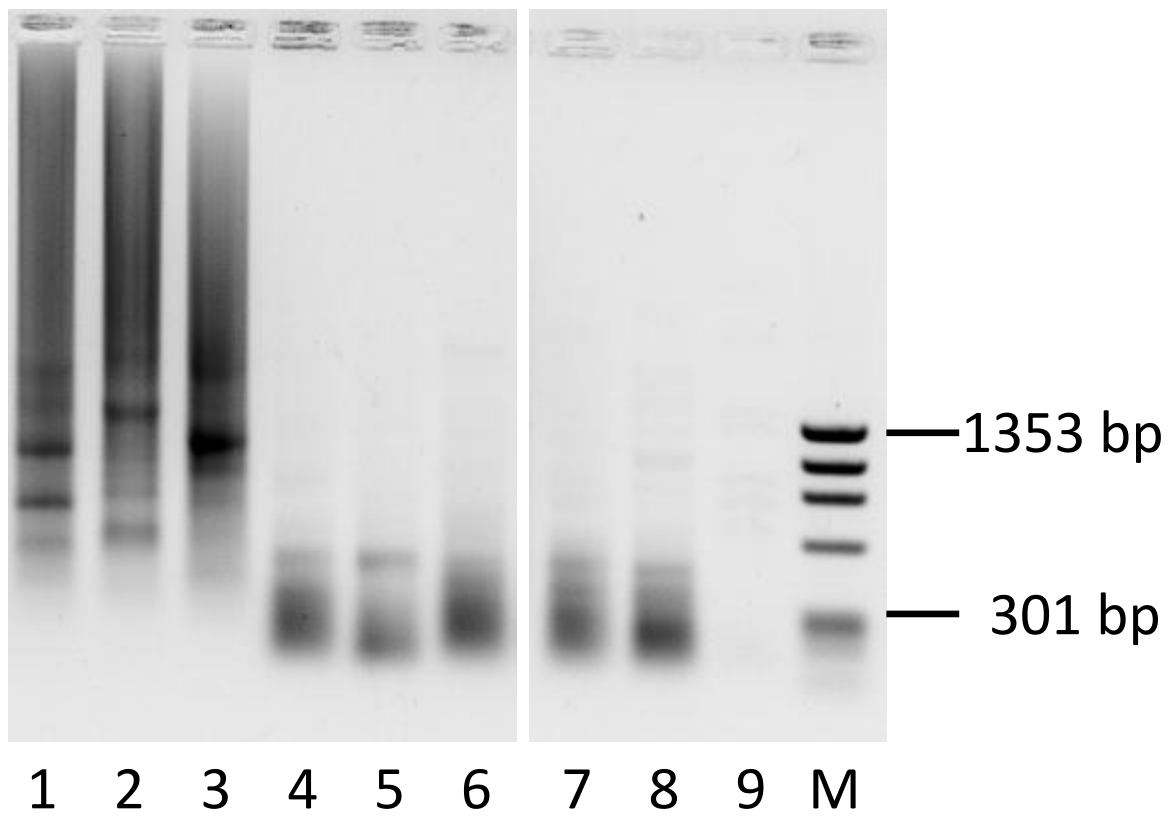

Supplement: Fig. S1 — Representative ethidium bromide-stained agarose gel showing randomly amplified DNA from poliovirus vaccines. (1–3) live monovalent Sabin vaccines, (4–6) inactivated monovalent Sabin vaccines, (7) water extraction control, (8) water control for cDNA synthesis, (9) water control for PCR, M: PhiX/HaeIII digest DNA size ladder. Gel has been cropped to remove unrelated samples. [file mmc1.pdf]

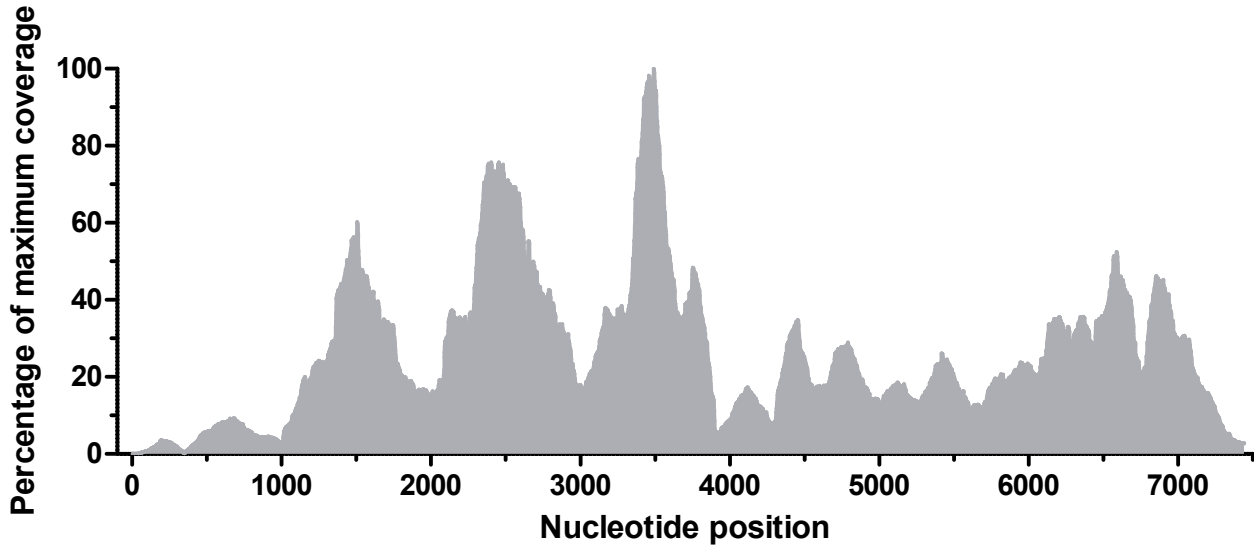

Supplement: Fig. S2 — Genome coverage of Sabin 1 in contaminated sIPV product. [file mmc2.pdf]
